# Upadacitinib monotherapy versus methotrexate monotherapy in patients with rheumatoid arthritis: efficacy and safety through 5 years in the SELECT-EARLY randomized controlled trial

**DOI:** 10.1186/s13075-024-03358-x

**Published:** 2024-07-29

**Authors:** Ronald van Vollenhoven, Vibeke Strand, Tsutomu Takeuchi, Nilmo Chávez, Pablo Mannucci Walter, Atul Singhal, Jerzy Swierkot, Nasser Khan, Xianwei Bu, Yihan Li, Sara K. Penn, Heidi S. Camp, Jacob Aelion

**Affiliations:** 1 Amsterdam University Medical Centers, Amsterdam, Netherlands; 2 Division Immunology/Rheumatology, Stanford University, Palo Alto, CA USA; 3 Keio University School of Medicine, Tokyo, Japan; 4 Saitama Medical University, Saitama, Japan; 5 Instituto Guatemalteco de Seguridad Social, Ciudad de Guatemala, Guatemala; 6 Aprillus Asistencia e Investigación, Buenos Aires, Argentina; 7 Southwest Rheumatology Research Group, Dallas, TX USA; 8 Department of Rheumatology and Internal Medicine, Wroclaw Medical University, Wroclaw, Poland; 9 AbbVie Inc, North Chicago, IL USA; 10 West Tennessee Research Institute, Jackson, TN USA

**Keywords:** JAK inhibitor, Methotrexate, Targeted synthetic DMARD, Upadacitinib, Rheumatoid arthritis, Randomized controlled trial, Long-term extension, SELECT-EARLY

## Abstract

**Background:**

To evaluate the efficacy and safety of upadacitinib monotherapy versus methotrexate (MTX) monotherapy over 5 years among MTX-naïve patients with moderately to severely active rheumatoid arthritis (RA) in the long-term extension (LTE) of the phase 3 SELECT-EARLY trial.

**Methods:**

Patients were randomized to receive upadacitinib 15 mg or 30 mg or MTX. Patients who did not achieve CDAI remission and had < 20% improvement in tender and swollen joint counts at week 26 received rescue therapy (addition of MTX in the upadacitinib group and addition of upadacitinib in the MTX group). Efficacy assessments were evaluated over 5 years and are reported as observed (AO) for patients who received continuous monotherapy with upadacitinib 15/30 mg or MTX and by randomized group applying non-responder imputation (NRI). Treatment-emergent adverse events (TEAEs) per 100 patient-years were summarized over 5 years.

**Results:**

Of 945 patients randomized and treated, 775 (82%) completed week 48 and entered the LTE on study drug. Higher proportions of patients consistently achieved disease activity targets over 5 years with upadacitinib than MTX. In AO analyses, 53%/59% of patients attained CDAI remission with upadacitinib 15/30 mg versus 43% with MTX at week 260. NRI analyses showed better CDAI, DAS28(CRP), and ACR responses with upadacitinib relative to MTX at week 260 (all comparisons, nominal *P* < .001). Upadacitinib treatment also resulted in numerically greater inhibition of structural joint progression through week 260 compared to MTX. Most TEAEs, serious AEs, and AEs leading to discontinuation were numerically higher in patients receiving upadacitinib 30 mg. Rates of serious infections, herpes zoster, creatine phosphokinase elevation, nonmelanoma skin cancer, and neutropenia were numerically higher with upadacitinib than MTX. The observed safety profile of upadacitinib over 5 years was consistent with earlier trial results and integrated phase 3 safety analyses.

**Conclusions:**

Upadacitinib showed better clinical responses versus MTX in patients with RA throughout the 5-year trial. Higher rates of several AEs were observed with upadacitinib, especially in the 30 mg group, compared to MTX. When used as monotherapy in MTX-naïve patients, the approved upadacitinib 15 mg dose showed better long-term efficacy versus MTX and an overall favorable benefit-risk profile.

**Trial registration:**

NCT02706873.

**Supplementary Information:**

The online version contains supplementary material available at 10.1186/s13075-024-03358-x.

## Introduction

Rheumatoid arthritis (RA) is a systemic inflammatory disease that can cause impaired functional ability, chronic pain, and increased mortality rates if not properly treated [1]. Methotrexate (MTX) is the most commonly used first-line therapeutic choice for RA due to its well-known safety profile and demonstrated effectiveness in reducing disease activity and preventing joint damage [2–4]. However, not all patients respond well to MTX or can tolerate its side effects, highlighting the need for alternative therapeutic options [5–7]. Inhibition of Janus kinase (JAK)-mediated pathways has emerged as one such alternative mechanism of action for the treatment of RA [8, 9].

The JAK inhibitor upadacitinib has been extensively evaluated as part of the phase 3 SELECT RA clinical program, which involves six trials comprised of approximately 4800 patients with moderately to severely active RA, including those who have not responded adequately to prior conventional synthetic (cs) disease-modifying antirheumatic drugs (DMARDs) or biologic DMARDs (bDMARDs) [8, 10–15]. In the SELECT-EARLY trial, which focused on MTX-naïve patients, once daily treatment with upadacitinib 15 mg or 30 mg as monotherapy led to significant improvements in clinical, functional, and patient-reported outcomes over a 24-week period compared to MTX monotherapy [12]. In terms of safety, the rates of adverse events were slightly higher with upadacitinib 30 mg compared to upadacitinib 15 mg or MTX through 24 weeks. However, considering the chronic nature of RA and ongoing treatment requirements for most patients, it is crucial to assess the long-term safety of any therapeutic intervention. Thus, our objective in this analysis was to examine the efficacy and safety of upadacitinib over a 5-year period in the long-term extension (LTE) of the SELECT-EARLY trial.

## Methods

### Patients

Study eligibility criteria and baseline demographics for SELECT-EARLY have been previously described [12]. In brief, patients were ≥ 18 years old, exhibited symptoms consistent with RA for ≥ 6 weeks, and fulfilled the 2010 American College of Rheumatology (ACR)/European Alliance of Associations for Rheumatology (EULAR) classification criteria for RA. Additional eligibility criteria included ≥ 6 swollen joints (based on 66 joint counts), ≥ 6 tender joints (based on 68 joint counts), and high-sensitivity C-reactive protein ≥ 5 mg/L (upper limit of normal 2.87 mg/L). Eligible patients also had ≥ 1 bone erosion on radiography or both positive rheumatoid factor (RF) and positive anti–citrullinated protein antibodies (ACPA) at screening. Patients were naïve to MTX, or if already on MTX, had received ≤ 3 weekly MTX doses with a requirement to complete a 4-week MTX washout before the first dose of study drug. Patients with prior exposure to csDMARDs other than MTX may have been enrolled if they completed a pre-defined washout period. Patients were excluded if they were intolerant to MTX, had prior exposure to any JAK inhibitor or any bDMARD, had a history of any arthritis with onset prior to age 17 years, or a current diagnosis of inflammatory joint disease other than RA.

The study was conducted in accordance with the International Conference on Harmonisation of Technical Requirements for Pharmaceuticals for Human Use guidelines, applicable regulations, and the Declaration of Helsinki. All study-related documents were approved by independent ethics committees and institutional review boards. All patients provided written informed consent.

### Study design and treatments

SELECT-EARLY (clinical trial number: NCT02706873) included a 48-week double-blind treatment period followed by an open-label LTE period for up to an additional four years (Supplemental Fig. 1). Patients were randomized 1:1:1 to receive once daily upadacitinib 15 mg or 30 mg or MTX (titrated up to 20 mg/week by week 8). A Japan substudy was also conducted in which patients were randomized 2:1:1:1 to receive once daily upadacitinib 7.5 mg, 15 mg, 30 mg or MTX; however, data from the 7.5 mg dose are not presented here. Treatment with background non-steroidal anti-inflammatory drugs (NSAIDs), acetaminophen, oral glucocorticoids (equivalent to prednisone ≤ 10 mg/day), or inhaled glucocorticoids was allowed but must have been at a stable dose ≥ 1 week prior to the first dose of study drug. Optimization of some background RA medications was permitted from week 12 to week 24 (including NSAIDs, glucocorticoids, and/or low-potency analgesics). At week 26, patients who did not achieve Clinical Disease Activity Index (CDAI) remission (≤ 2.8) and at least 20% improvement from baseline in tender and swollen joint counts received rescue therapy (addition of MTX in the upadacitinib groups and addition of upadacitinib 15/30 mg [by re-randomization] in the MTX group). From week 36 to week 40, further optimization of background RA medications was allowed (including NSAIDs, glucocorticoids, low-potency analgesics, and/or csDMARD [only 1 of the following: sulfasalazine, hydroxychloroquine, or chloroquine]). Starting at week 48, initiation of or change in background RA medication(s), including glucocorticoids, NSAIDs, acetaminophen/paracetamol, and csDMARDs (≤ 2 csDMARDs except the combination of MTX and leflunomide) was allowed according to local label. Per protocol amendment, all patients receiving upadacitinib 30 mg were later switched to the approved 15 mg dose. Most patients who received upadacitinib 30 mg switched to 15 mg between weeks 158 to 192, with the earliest switch occurring at week 108.

### Efficacy assessments

Efficacy assessments included the proportions of patients attaining clinical remission (defined by CDAI ≤ 2.8) or low disease activity (LDA; defined by CDAI ≤ 10) [16], 28-joint Disease Activity Score based on C-reactive protein (DAS28(CRP) < 2.6 or ≤ 3.2) [17, 18], ACR20/50/70 response criteria [19], and the 2010 definition of ACR/EULAR Boolean remission [20]. Additionally, changes from baseline in ACR components such as Health Assessment Questionnaire-Disability Index (HAQ-DI) [21] and patient’s assessment of pain were examined, along with the severity and duration of morning stiffness. Radiographic assessments were completed at weeks 24, 96, 192, and 260 and included the proportion of patients with no radiographic progression (defined as modified Total Sharp Score [mTSS] ≤ 0) [22, 23] and change from baseline in mTSS, joint space narrowing, and erosion scores. All other efficacy assessments were performed every 12 weeks during the LTE.

### Safety assessments

Data on safety outcomes were collected from all patients who received upadacitinib or MTX, with adverse event (AE) assignment determined based on the treatment at the time of the event. All treatment-emergent adverse events (TEAEs) were summarized using the Medical Dictionary for Regulatory Activities (MedDRA) (version 25.0) primary system organ class and preferred term. TEAEs were defined as any events that occurred after the first dose of study drug, but no later than 30 days after the last dose of study drug. All presented AEs were treatment-emergent, with the exception of mortality assessments, which also included deaths that were not treatment-emergent and occurred more than 30 days after the last dose of study drug.

Safety assessments were performed as previously described [12]. Major adverse cardiovascular events (MACE) and venous thromboembolism (VTE) events were adjudicated by an independent Cardiovascular Adjudication Committee (CAC) in a blinded manner. MACE were defined as either cardiovascular (CV) death, non-fatal myocardial infarction (MI), or non-fatal stroke. VTE was defined as deep vein thrombosis (DVT) or pulmonary embolism (PE). Gastrointestinal (GI) perforations were adjudicated by an internal independent committee of gastroenterologists. Laboratory parameters were assessed up to week 260, including the percentage of patients experiencing potentially clinically significant (grade 3 or 4) changes during the treatment period. Per protocol, study investigators evaluated the severity of TEAEs according to the Rheumatology Common Toxicity Criteria (CTC) version 2.0 (Outcome Measures in Rheumatology [OMERACT]). For creatine phosphokinase (CPK) and creatinine, however, National Cancer Institute (NCI) CTC version 4.0 criteria were used.

### Statistical analysis

Efficacy assessments were evaluated through 5 years and are reported as observed (AO) for patients who received continuous monotherapy with upadacitinib 15 mg or 30 mg or MTX. Efficacy outcomes are also reported by randomized group for all randomized and dosed study participants, applying NRI for patients who were rescued or discontinued. Treatment comparisons for NRI analyses were made using the Cochran-Mantel-Haenszel test, adjusting for the stratification factor of geographic region. Continuous endpoints were evaluated using AO and the mixed model for repeated measures (MMRM) analysis for patients receiving continuous monotherapy. Given the small sample size of patients who received rescue therapy (i.e., upadacitinib 15 mg or 30 mg plus MTX), efficacy is not reported separately for these patients. For inclusion in radiographic analyses, patients were required to have X-ray images at both week 192 and week 260. Data collected after dose switch from upadacitinib 30 mg to the approved 15 mg dose following protocol amendment continued to be summarized under the original treatment sequences, regardless of dose switch. A separate evaluation of CDAI responses before and after the dose switch was also conducted.

TEAEs per 100 patient-years were summarized through 5 years for patients receiving either dose of upadacitinib monotherapy or MTX monotherapy, as well as patients who switched from upadacitinib monotherapy at 30 mg to 15 mg for the post-switch period. Upadacitinib or MTX monotherapy exposure was censored at time of rescue to upadacitinib plus MTX/csDMARD. In addition, upadacitinib 30 mg exposure was censored at the time of dose switch from 30 mg to 15 mg. All safety data are reported as exposure-adjusted event rates (EAERs), defined as events per 100 patient-years (E/100 PY).

## Results

### Patients

**Fig. 1:**
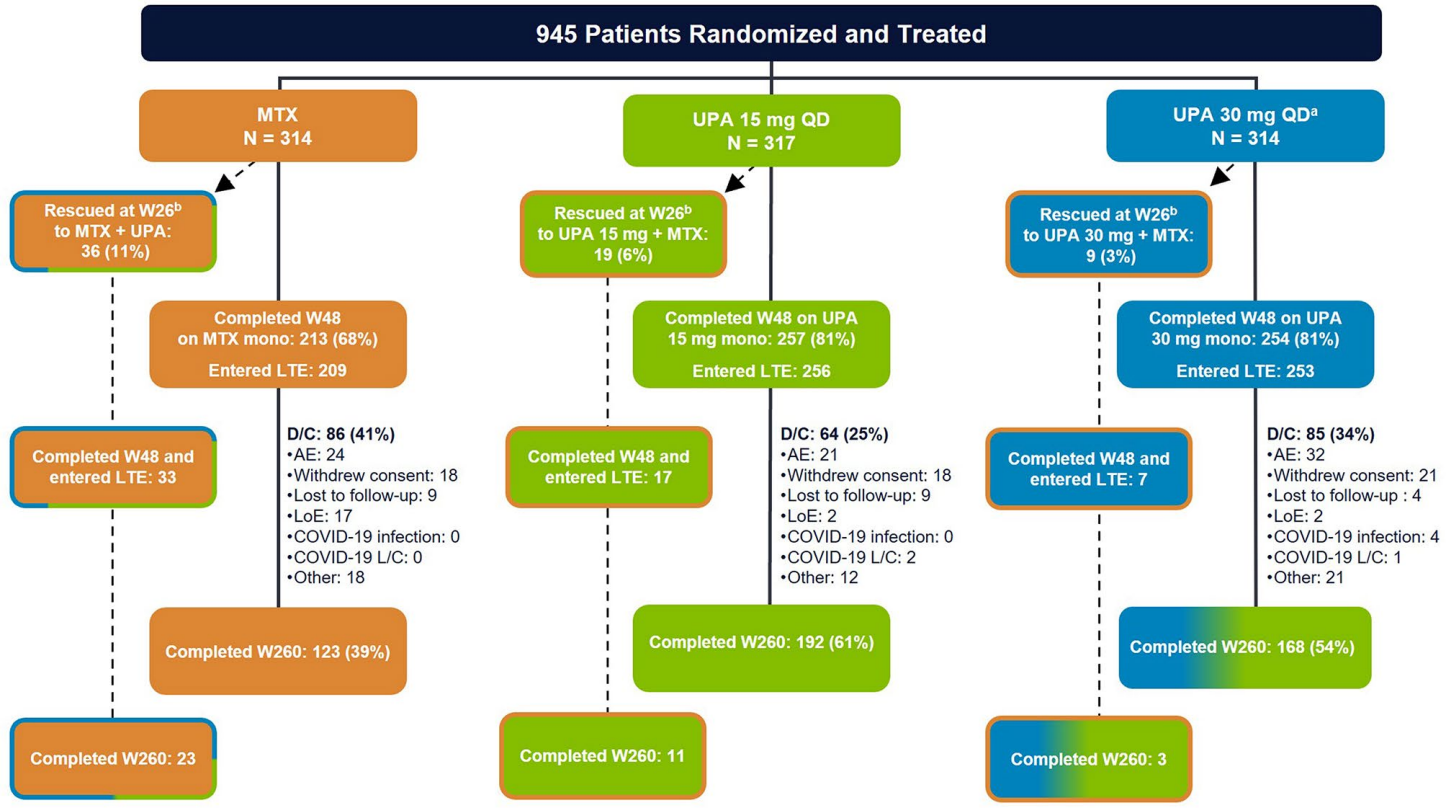
Disposition of Patients Through 5 Years in SELECT-EARLY. AE, adverse event; CDAI, Clinical Disease Activity Index; D/C, discontinued; L/C, logistical constraints; LoE, lack of efficacy; MTX, methotrexate; QD, once daily; UPA, upadacitinib; W, week. ^a^Patients in the UPA 30 mg treatment group were later switched to the approved UPA 15 mg dose per protocol amendment. The switch occurred at different visits across the patient population, with the earliest switch occurring at the week 108 visit. ^b^At week 26, patients who did not achieve CDAI remission and at least 20% improvement from baseline in tender and swollen joint counts received rescue therapy (addition of MTX to insufficient responders in the UPA groups and addition of UPA 15/30 mg to insufficient responders in the MTX group)

Baseline characteristics were generally balanced across all treatment groups (Supplemental Table 1), as previously reported [12]; 75% of patients had no exposure to any csDMARD prior to enrollment. Most patients had early RA (median disease duration of 0.5 years) and risk factors for structural progression, as demonstrated by positivity for RF and ACPA and/or at least one bone erosion. Of the 945 patients randomized and treated, 775 (82%) completed week 48 and entered the LTE on study drug (Fig. 1). Of these 775 patients, 255 (33%) discontinued study drug during the LTE. The most common primary reason for discontinuation was TEAE (11%), followed by withdrawal of consent (8%), lack of efficacy (3%), lost to follow-up (3%), or other reasons (8%). Higher proportions of patients randomized to MTX (11%) received rescue therapy versus those who initially received either dose of upadacitinib (3–6%). Similarly, higher proportions of patients randomized to upadacitinib 15 mg (61%) or 30 mg (54%) completed 5 years of continuous treatment relative to those who started with MTX (39%).

During the study, approximately half of patients used concomitant glucocorticoids (55%, 53%, and 59% for upadacitinib 15 mg, upadacitinib 30 mg, and MTX monotherapy groups, respectively). Among patients with glucocorticoid use at baseline, the percentages of patients who discontinued their use by week 260 were as follows: 36% (*n* = 38/107) for upadacitinib 15 mg, 48% (*n* = 41/86) for upadacitinib 30 mg, and 39% (*n* = 28/71) for MTX.

### Efficacy

**Fig. 2:**
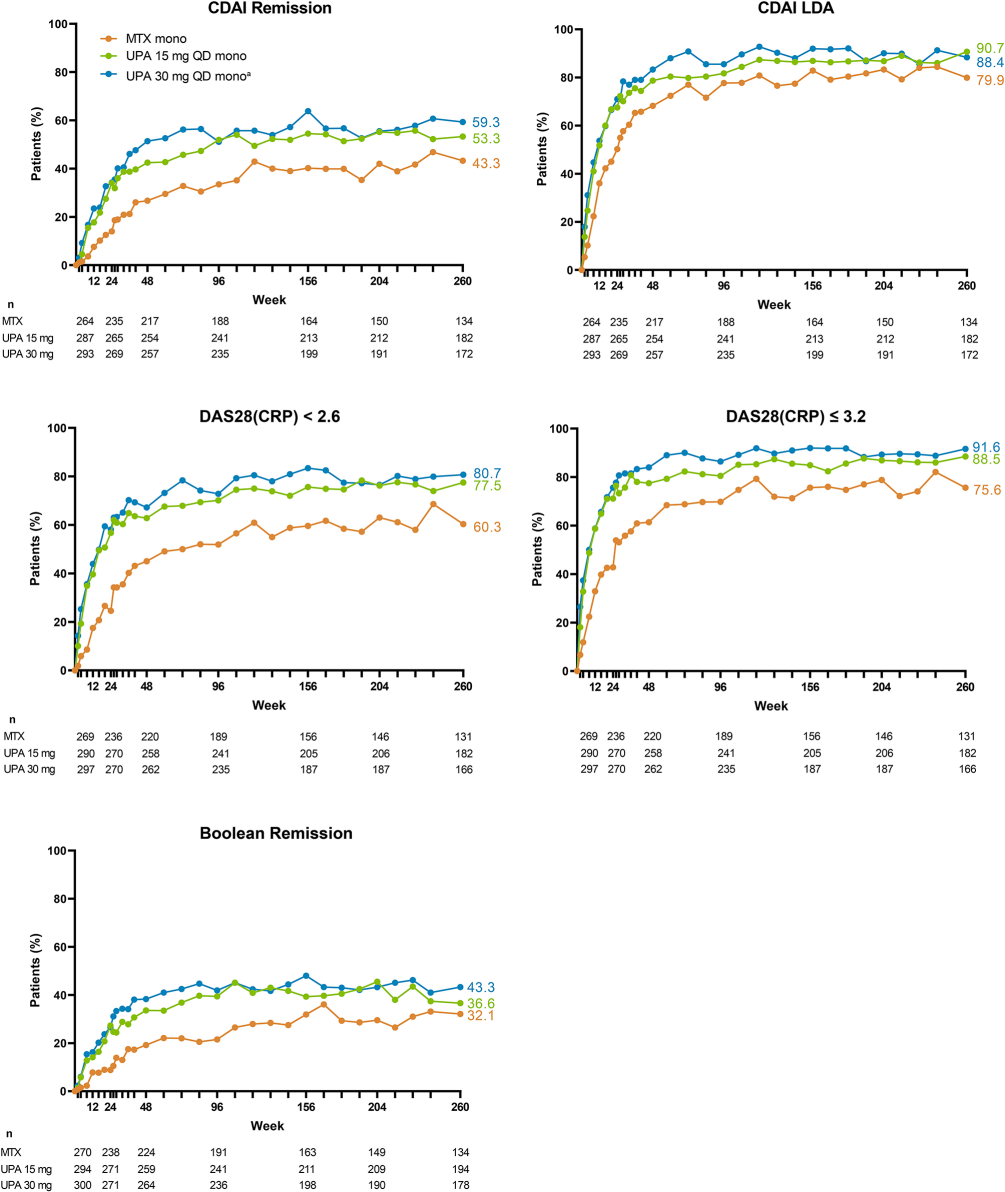
Proportions of Patients Achieving CDAI, DAS28(CRP) Disease Activity States and Boolean Remission Through 5 Years (AO). AO, as observed; CDAI, Clinical Disease Activity Index; DAS28(CRP), 28-joint Disease Activity Score based on C-reactive protein; LDA, low disease activity; mono, monotherapy; MTX, methotrexate; QD, once daily; UPA, upadacitinib. ^a^Patients in the UPA 30 mg treatment group were later switched to the approved UPA 15 dose mg per protocol amendment. The switch occurred at different visits across the patient population, with the earliest switch occurring at the week 108 visit. Thresholds for CDAI were ≤ 2.8 for remission and ≤ 10 for LDA

**Fig. 3:**
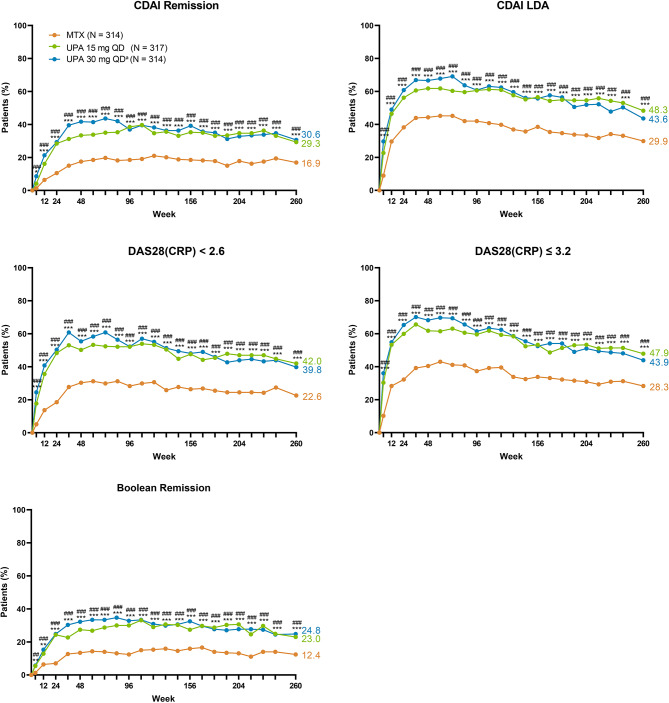
Proportions of Patients Achieving CDAI, DAS28(CRP) Disease Activity States and Boolean Remission Through 5 Years (NRI). CDAI, Clinical Disease Activity Index; DAS28(CRP), 28-joint Disease Activity Score based on C-reactive protein; LDA, low disease activity; MTX, methotrexate; NRI, non-responder imputation; QD, once daily; UPA, upadacitinib. ^a^Patients in the UPA 30 mg treatment group were later switched to the approved UPA 15 mg dose per protocol amendment. The switch occurred at different visits across the patient population, with the earliest switch occurring at the week 108 visit. Indicated assessments from period 1 (at weeks 4, 12, 24, 36, and 48) are summarized here along with all assessments from the long-term extension. Nominal ^***^P < .001, ^**^P < .01, and ^*^P < .05 for upadacitinib 15 mg versus MTX. Nominal ^###^*P* < .001, ^##^*P* < .01, and ^#^*P* < .05 for upadacitinib 30 mg versus MTX. Thresholds for CDAI were ≤ 2.8 for remission and ≤ 10 for LDA

Patients receiving upadacitinib consistently demonstrated higher achievement of disease activity targets over 5 years compared with those receiving MTX. In AO analyses, 53%/59% of patients attained CDAI remission with upadacitinib 15/30 mg monotherapy versus 43% with MTX monotherapy at week 260 (Fig. 2). Similarly, 91%/88% achieved CDAI LDA with upadacitinib 15/30 mg compared to 80% with MTX at week 260; attainment of DAS28(CRP) < 2.6 and ≤ 3.2 with upadacitinib 15/30 mg was 78%/81% and 89%/92% compared to 60% and 76% with MTX, respectively (AO; Fig. 2). Among patients randomized to upadacitinib 30 mg, those who switched to the approved 15 mg dose, per protocol amendment, maintained their CDAI responses (Supplemental Fig. 2). NRI analyses also showed better CDAI and DAS28(CRP) responses with upadacitinib 15/30 mg relative to MTX at week 260 (all comparisons from week 8 to week 260, nominal *P* < .001) (Fig. 3). Higher proportions of patients also attained the stringent Boolean-based definition of remission with upadacitinib compared to MTX (Figs. 2 and 3). Based on NRI analyses at week 260, 23%/25% of patients attained Boolean remission with upadacitinib 15/30 mg treatment versus 12% with MTX (both comparisons, nominal *P* < .001 (Fig. 3).

**Fig. 4:**
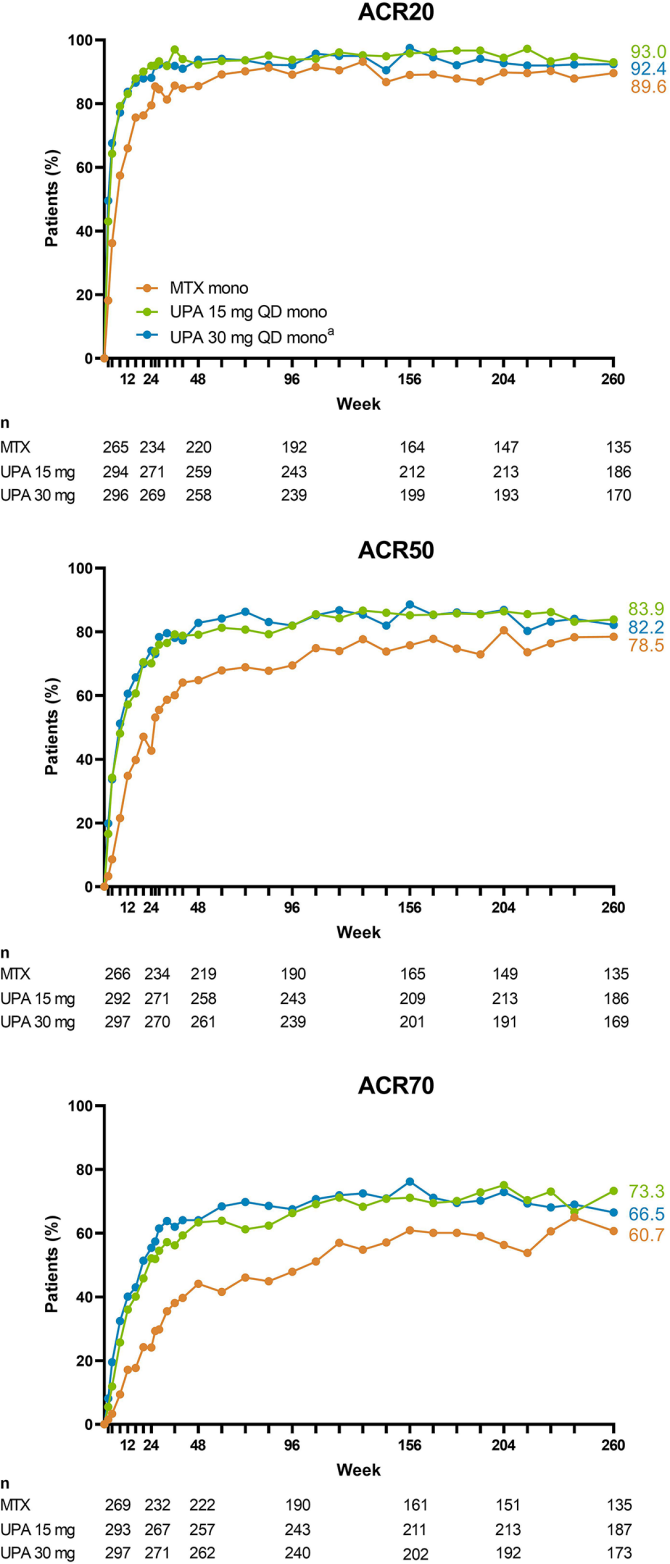
Proportions of Patients Achieving ACR Response Criteria Through 5 Years (AO). ACR20/50/70, ≥ 20%/50%/70% improvement in American College of Rheumatology response criteria; AO, as observed; mono, monotherapy; MTX, methotrexate; QD, once daily; UPA, upadacitinib. ^a^Patients in the UPA 30 mg treatment group were later switched to the approved UPA 15 mg dose per protocol amendment. The switch occurred at different visits across the patient population, with the earliest switch occurring at the week 108 visit

**Fig. 5:**
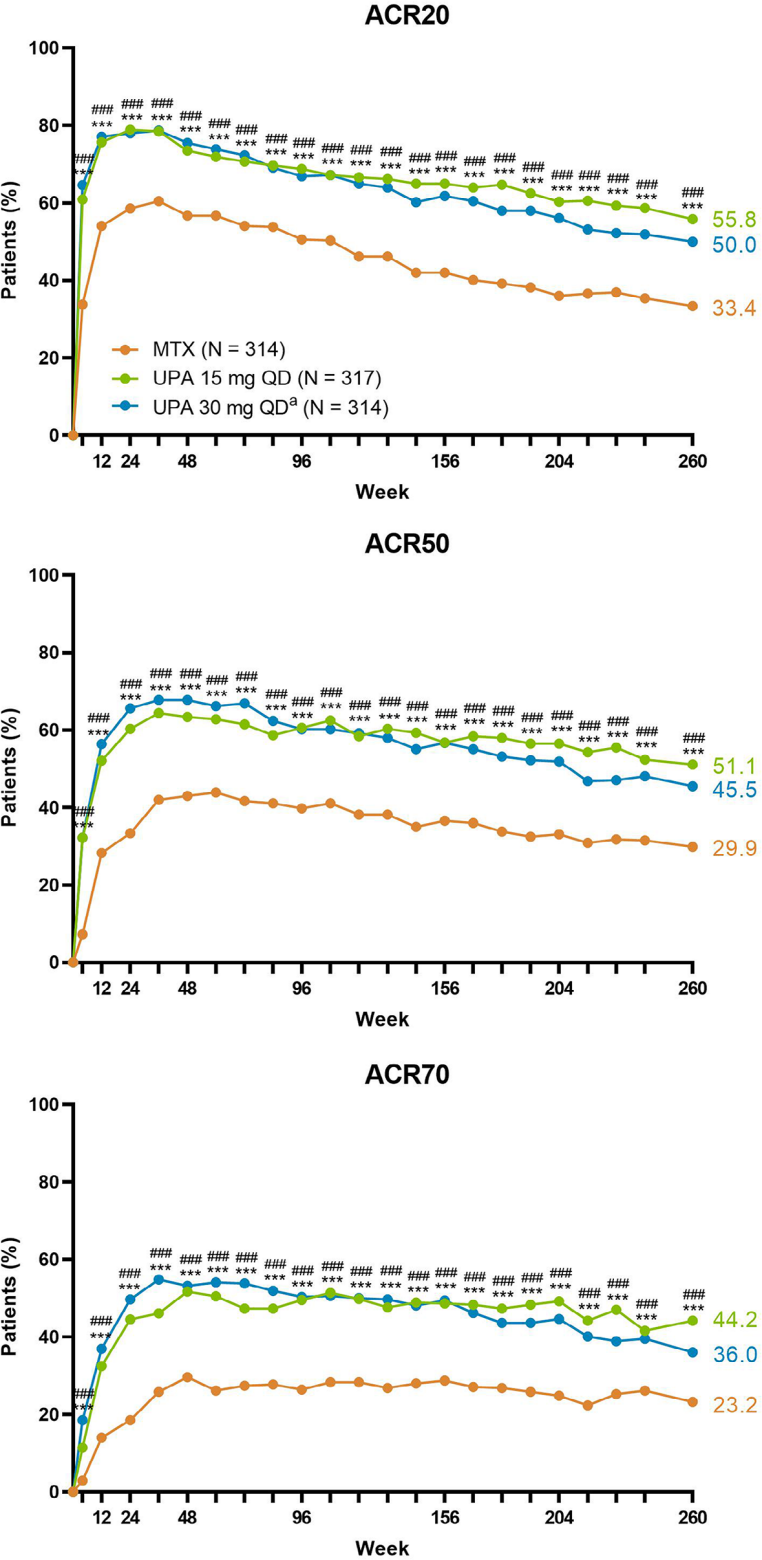
Proportions of Patients Achieving ACR Response Criteria Through 5 Years (NRI). ACR20/50/70, ≥ 20%/50%/70% improvement in American College of Rheumatology response criteria; mono, monotherapy; MTX, methotrexate; NRI, non-responder imputation; QD, once daily; UPA, upadacitinib. ^a^Patients in the UPA 30 mg treatment group were later switched to the approved UPA 15 mg dose per protocol amendment. The switch occurred at different visits across the patient population, with the earliest switch occurring at the week 108 visit. Indicated assessments from period 1 (at weeks 4, 12, 24, 36, and 48) are summarized here along with all assessments from the long-term extension. Nominal ^***^P < .001, ^**^P < .01, and ^*^P < .05 for upadacitinib 15 mg versus MTX. Nominal ^###^*P* < .001, ^##^*P* < .01, and ^#^*P* < .05 for upadacitinib 30 mg versus MTX

Numerically greater proportions of patients achieved ACR20/50/70 responses with upadacitinib 15 mg or 30 mg compared to MTX over 5 years (AO; Fig. 4). When analyzing the results by NRI, more patients randomized to upadacitinib 15 mg or 30 mg met ACR20/50/70 criteria at week 260 than those randomized to MTX (upadacitinib 15 mg: 56%/51%/44%; upadacitinib 30 mg: 50%/46%/36%; MTX: 33%/30%/23%; all comparisons, nominal *P* < .001) (Fig. 5). Additionally, patients receiving upadacitinib 15 mg or 30 mg demonstrated greater numerical improvements from baseline in HAQ-DI and pain at week 260 compared to those treated with MTX monotherapy (HAQ-DI: -1.13/-1.03 with upadacitinib 15/30 mg versus − 0.89 with MTX; pain: -51.2/-49.1 with upadacitinib 15/30 mg versus − 41.9 with MTX; AO) (Supplemental Fig. 3). Consistent results were observed for other ACR core components over 5 years, except for 68-tender joint count, 66-swollen joint count, and physician’s global assessment in which responses were generally similar between groups. Treatment with both upadacitinib doses also led to improvements in morning stiffness relative to MTX (Supplemental Fig. 4).

**Fig. 6:**
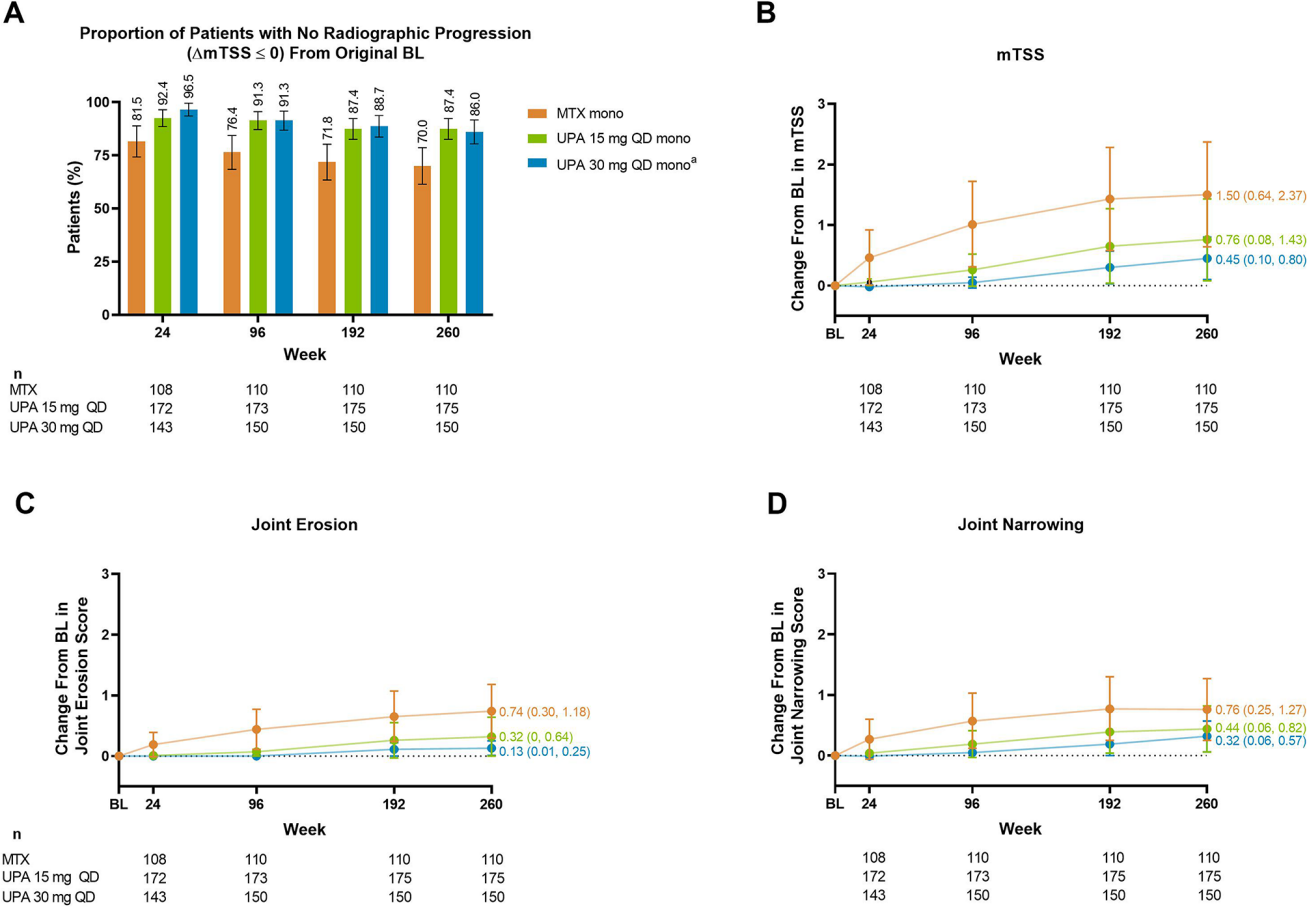
Radiographic Progression Through 5 Years (AO). AO, as observed; BL, baseline; mono, monotherapy; mTSS, modified Total Sharp Score; MTX, methotrexate; QD, once daily; UPA, upadacitinib. ^a^Patients in the UPA 30 mg treatment group were later switched to the approved UPA 15 mg dose per protocol amendment. The switch occurred at different visits across the patient population, with the earliest switch occurring at the week 108 visit. Patients were required to have radiographic images at both week 192 and week 260 for inclusion in these analyses. Error bars represent the 95% confidence interval

Regarding structural joint damage, 87%/86% of patients receiving upadacitinib 15/30 mg monotherapy had no radiographic progression (defined as a change from baseline in mTSS ≤ 0) at week 260 compared to 70% of those receiving MTX monotherapy (Fig. 6). The change from baseline in mTSS, erosion score, and joint space narrowing was numerically lower with upadacitinib 15 mg and 30 mg relative to MTX at all radiographic assessments through week 260.

### Safety

The mean duration of exposure was 3.4 years with upadacitinib 15 mg monotherapy, 2.4 years with upadacitinib 30 mg monotherapy, and 2.7 years with MTX monotherapy. Most TEAEs, including serious AEs, were more frequent in patients receiving upadacitinib 30 mg monotherapy than those receiving upadacitinib 15 mg or MTX monotherapy, and patients who switched from upadacitinib 30 mg to the approved 15 mg dose per protocol amendment had similar event rates post-switch compared to those initially randomized to upadacitinib 15 mg (Table 1). Across treatment groups, the majority of TEAEs (~ 95%) were mild to moderate in severity. The rates of TEAEs leading to discontinuation of study drug were comparable in the upadacitinib 15 mg group and the MTX group, but higher in the upadacitinib 30 mg group. The most frequently reported TEAEs (≥ 5 E/100 PY in any dose group) for patients receiving upadacitinib were elevated CPK, upper respiratory tract infection, and urinary tract infection (Supplemental Table 2).

**Fig. 7:**
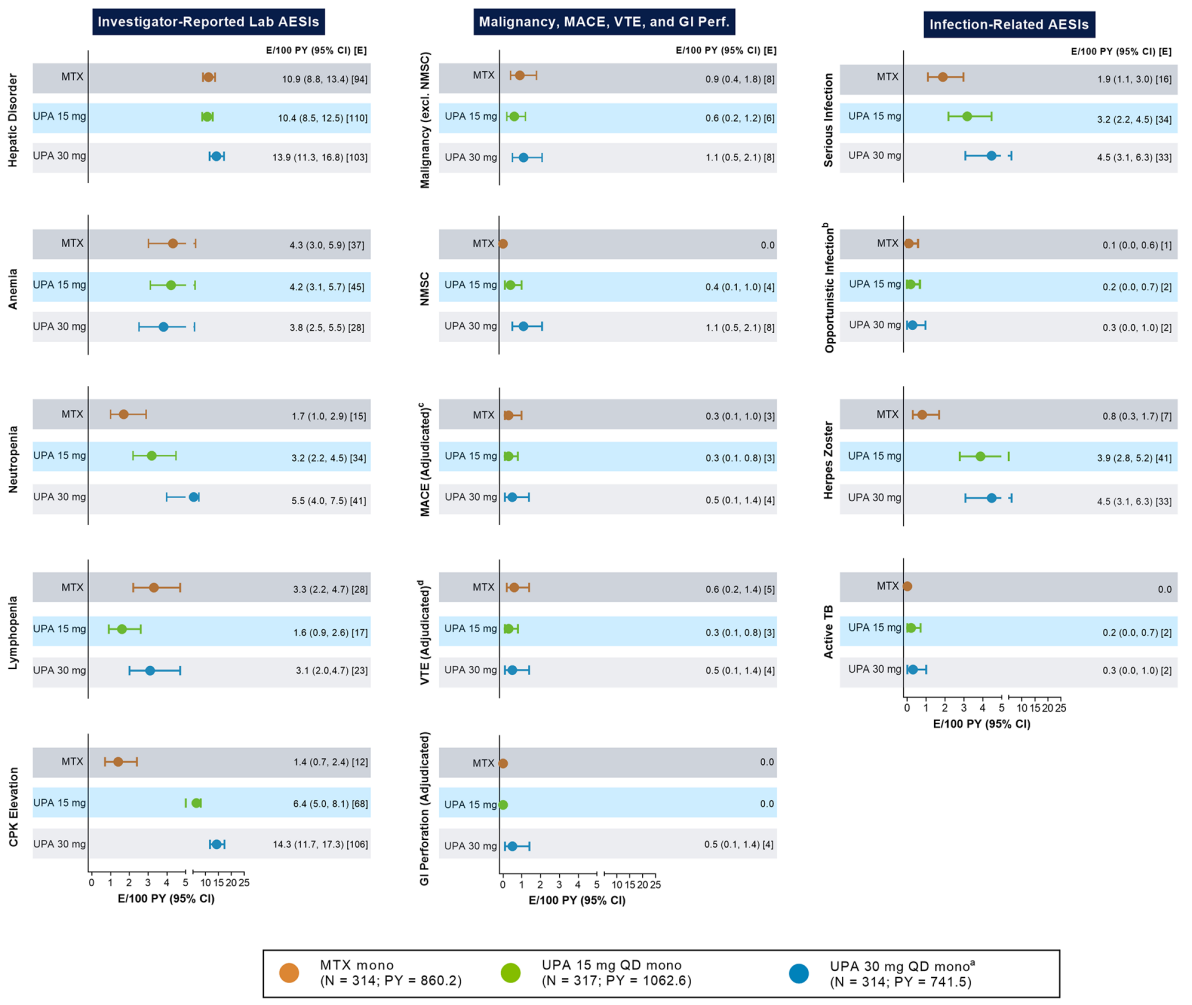
Exposure-Adjusted Event Rates for Adverse Events of Special Interest Through 5 Years. AE, adverse event; AESI, adverse event of special interest; CPK, creatine phosphokinase; DMARD, disease-modifying antirheumatic drug; GI, gastrointestinal; MACE, major adverse cardiovascular event; mono, monotherapy; MTX, methotrexate; NMSC, nonmelanoma skin cancer; PY, patient-years; QD, once daily; TB, tuberculosis; UPA, upadacitinib; VTE, venous thromboembolism. Treatment-emergent adverse event is defined as any adverse event with an onset date that is after the first dose of study drug, and no more than 30 days after the last dose of study drug. Data include patients receiving UPA or MTX monotherapy, censored at either time of rescue to UPA + MTX or with addition of background conventional synthetic DMARD. ^a^Patients in the UPA 30 mg treatment group were later switched to the approved UPA 15 mg dose per protocol amendment. Upadacitinib 30 mg QD exposure was censored at the time of dose switch from 30 mg QD to 15 mg QD. ^b^Opportunistic infections exclude herpes zoster and TB. ^c^Defined as cardiovascular death, non-fatal myocardial infarction, and non-fatal stroke. ^d^Includes pulmonary embolism and deep vein thrombosis

Adverse events of special interest (AESIs) were consistent with the established safety profile of upadacitinib, and no new safety issues were identified from the long-term study. The most frequently reported AESIs (> 5 E/100 PY in any treatment group) were hepatic disorder, neutropenia, and CPK elevation (Fig. 7). EAERs of CPK elevation, neutropenia, serious infections, herpes zoster, and nonmelanoma skin cancer (NMSC) were numerically higher with upadacitinib 15 mg or 30 mg than MTX. Rates of CPK elevation appeared to be dose-dependent with upadacitinib (6.4 and 14.3 E/100 PY for upadacitinib 15 mg and 30 mg groups, respectively) and lower with MTX (1.4 E/100 PY). Most CPK events were mild to moderate in severity, asymptomatic, and transient; there were no reports of rhabdomyolysis. Two events of CPK elevation were serious (one each in the upadacitinib 15 mg and 30 mg plus MTX rescue group), one of which led to discontinuation of study drug. Rates of neutropenia were higher in the upadacitinib 30 mg monotherapy group (5.5 E/100 PY) than in the upadacitinib 15 mg monotherapy group (3.2 E/100 PY); rates in both upadacitinib groups were higher than in the MTX monotherapy group (1.7 E/100 PY). Most cases of neutropenia were mild to moderate in severity.

**Table 1:** Overview of adverse events through 5 years

Events (E/100 PY)^a^	MTX mono (*N* = 314; PY = 860.2)	UPA 15 mg QD mono (*N* = 317; PY = 1062.6)	UPA 30 mg QD mono^b^ (*N* = 314; PY = 741.5)	UPA 15 mg QD mono switched from UPA 30 mg QD mono (*N* = 181; PY = 292.5)
Any AE	1767 (205.4)	2396 (225.5)	2077 (280.1)	451 (154.2)
Serious AEs	78 (9.1)	111 (10.4)	118 (15.9)	44 (15.0)
Any AE leading to discontinuation of study drug	50 (5.8)	58 (5.5)	57 (7.7)	10 (3.4)
COVID-19	23 (2.7)	34 (3.2)	6 (0.8)	39 (13.3)
All deaths^c, d^	8 (0.9)	6 (0.6)	9 (1.2)	5 (1.7)
Deaths ≤ 30 days after last dose	1 (0.1)	3 (0.3)	8 (1.1)	3 (1.0)
Deaths > 30 days after last dose	7 (0.8)	3 (0.3)	1 (0.1)	2 (0.7)

DMARD, disease-modifying antirheumatic drug; E, event; mono, monotherapy; MTX, methotrexate; PY, patient-years; QD, once daily; TEAE, treatment-emergent adverse event; UPA, upadacitinib

^a^Except mortality, data are presented as treatment-emergent adverse events, which is defined as any adverse event with an onset date that is after the first dose of study drug and no more than 30 days after the last dose of study drug. Data include patients receiving UPA or MTX monotherapy, censored at either time of rescue to UPA + MTX or with addition of background conventional synthetic DMARD

^b^UPA 30 mg exposure was censored at time of dose switch to the approved 15 mg dose. Safety outcomes following the switch from UPA 30 mg to UPA 15 mg are reported separately (last column)

^c^Includes treatment-emergent and non-treatment-emergent deaths. Seven deaths were COVID-19-related: 2 on UPA 30 mg mono and 3 on UPA 15 mg mono switched from UPA 30 mg mono. Two COVID-19-related deaths also occurred in patients on MTX who switched from UPA 30 mg to UPA 15 mg (not shown in the table)

^d^In addition to the presented treatment groups, 1 death occurred in a patient receiving UPA 30 mg plus MTX

Four treatment-emergent adjudicated GI perforations were reported among three patients in the upadacitinib 30 mg monotherapy group, all of which were serious events, including diverticular perforation, peritonitis and gastric ulcer perforation, and large intestine perforation. Confounding factors for these patients included gastroesophageal reflux disease, hypothyroidism, osteoporosis, hypertension, gastritis, obesity, and diverticulosis; one patient was also a former smoker (1 pack per day for 40 years). No GI perforation events were reported in patients receiving upadacitinib 15 mg or MTX.

Consistent with the known increased risk of herpes zoster with JAK inhibitor treatment [24–26], rates of herpes zoster events were higher in the upadacitinib 15 mg and 30 mg monotherapy groups (3.9 E/100 PY and 4.5 E/100 PY, respectively) compared to the MTX monotherapy group (0.8 E/100 PY), with the highest rate in the upadacitinib 30 mg monotherapy group. Most herpes zoster events were nonserious and involved only 1 or 2 dermatomes. Five herpes zoster events were reported as having ophthalmic involvement (2 with upadacitinib 15 mg + MTX, 1 with upadacitinib 30 mg monotherapy, and 2 in the upadacitinib 30 mg switched to upadacitinib 15 mg monotherapy group). One patient receiving upadacitinib 30 mg + MTX had herpes zoster meningitis, which led to discontinuation of study drug, and the patient subsequently recovered.

Rates of serious infection were higher with upadacitinib 15 mg (3.2 E/100 PY) and 30 mg (4.5 E/100 PY) compared to MTX (1.9 E/100 PY). Pneumonia was the most frequently reported serious infection among upadacitinib monotherapy and MTX monotherapy groups, except the upadacitinib 30 mg switched to 15 mg group, where COVID-19 pneumonia was the most frequently reported serious infection. Treatment-emergent COVID-19 infection or COVID-19-related AEs were comparable across the upadacitinib 15 mg monotherapy and MTX monotherapy groups. Consistent with the timing of the transition of patients to the approved 15 mg dose from the 30 mg dose with the onset and course of the COVID-19 pandemic, rates of COVID-19-related AEs were lower in patients receiving upadacitinib monotherapy and higher in the upadacitinib 30 mg switched to 15 mg group.

EAERs of MACE, VTE, and malignancy excluding NMSC were generally comparable across treatment groups (Fig. 7). Rates of MACE were 0.3, 0.3, and 0.5 E/100 PY in the upadacitinib 15 mg, 30 mg, and MTX monotherapy groups, respectively; rates of VTE were 0.6, 0.3, and 0.5 E/100 PY; rates of malignancies excluding NMSC were 0.9, 0.6, and 1.1 E/100 PY. Additional details regarding these AESIs are reported in the Supplemental Materials text. Most patients who experienced MACE had at least 1 CV risk factor at baseline. Among the 24 cases of malignancies excluding NMSC that were reported among monotherapy groups, there was no clear pattern in the types of malignancies that were observed and most occurred in a single patient. Although the rates of any malignancy other than NMSC were similar between treatment groups, the rate of NMSC was higher in the upadacitinib 30 mg monotherapy group compared to the upadacitinib 15 mg group (1.1 and 0.4 E/100 PY, respectively), and there were no events of NMSC in the MTX monotherapy group.

EAERs of anemia and hepatic disorders were also generally similar across treatment groups. Most cases of anemia were mild to moderate in severity, and the majority of hepatic disorders events were mild, asymptomatic transaminase elevations (further detailed in Supplemental Materials text). Rates of lymphopenia were numerically lower in the upadacitinib 15 mg group (1.6 E/100 PY) compared to upadacitinib 30 mg or MTX groups (3.1 and 3.3 E/100 PY, respectively). Most lymphopenia events had a mild to moderate severity.

Rates of death were similar between upadacitinib 15 mg and MTX (0.6 and 0.9 E/100 PY, respectively) but numerically higher with upadacitinib 30 mg (1.2 E/100 PY). Of the 31 deaths reported during the study, 8 were adjudicated by the CAC as cardiovascular in nature, 7 were COVID-19-related, and 4 had unknown/undetermined causes (Supplemental Materials text). All 7 COVID-19-related deaths occurred in patients treated with upadacitinib 15 mg or 30 mg. Common underlying risk factors included hypertension, obesity, tobacco use, and/or diabetes.

There were no notable mean changes in laboratory parameter values (hematology, clinical chemistry, and urinalysis) from baseline. However, higher proportions of patients with grade 3/4 CPK elevations were reported with upadacitinib than MTX, with the highest proportions occurring in the upadacitinib 30 mg monotherapy group (Supplemental Table 3). Among patients with an increase in blood CPK values that were grade 3/4, most were asymptomatic. The percentage of patients with a grade 3 decrease in neutrophils also occurred more frequently with either dose of upadacitinib than MTX; however, most instances were isolated events, occurring at only one time point.

## Discussion

The management of RA involves a multi-faceted approach, with the goals of sustained disease remission, or at least LDA, and prevention of long-term structural joint damage [3, 4]. MTX has long been considered the cornerstone of RA treatment, but the development of newer targeted therapies over the last decade has expanded the treatment options available to patients, particularly in those who do not respond adequately to MTX or are intolerant of its side effects. Our study provides valuable insights into the head-to-head efficacy and safety of upadacitinib monotherapy versus MTX monotherapy in patients with early RA and risk factors for structural joint progression. Throughout the 5-year study, upadacitinib 15 mg and 30 mg once daily continued to be effective in treating the signs and symptoms of RA, resulting in better long-term efficacy versus MTX. Higher rates of several AEs, including serious infection, herpes zoster, and CPK elevation were observed with upadacitinib, particularly in the 30 mg dose group, compared to MTX. No new safety issues emerged from the long-term study, and our findings are consistent with the known safety profile of upadacitinib [24, 27].

Although many patients were able to achieve disease activity targets with MTX monotherapy, as expected in this mostly (~ 93%) MTX naïve population, those receiving upadacitinib 15 mg or 30 mg monotherapy showed consistently better efficacy responses. In terms of clinical remission, the primary treatment target for this population, 53% and 59% of patients achieved CDAI remission with upadacitinib 15 mg and 30 mg, respectively, versus 43% with MTX (AO). Similarly, greater proportions of patients achieved CDAI LDA, DAS28(CRP), and ACR20/50/70 targets with either upadacitinib 15 mg or 30 mg versus MTX. These results are further supported by NRI analyses, indicating that upadacitinib 15 mg and 30 mg outperformed MTX across all evaluated efficacy endpoints at week 260 (all comparisons, nominal *P* < .001). Over the 5-year study, notable improvements from baseline were observed for physical function and pain across all groups, with upadacitinib 15 mg or 30 mg treatment resulting in numerically greater improvements relative to MTX. Morning stiffness, a key symptom of RA that can greatly impact a patient’s daily life and work [28], also showed reduced severity and duration with upadacitinib treatment compared to MTX. Additionally, there was no apparent loss of benefit in patients who switched from upadacitinib 30 mg to the approved 15 mg dose following protocol amendment.

Treatment with either upadacitinib or MTX demonstrated an inhibitory effect on structural joint damage in patients with RA. However, a larger percentage of patients receiving upadacitinib 15 mg and 30 mg monotherapy showed no radiographic progression, as determined by a change from baseline to week 260 in mTSS ≤ 0, compared to those receiving MTX monotherapy. Changes from baseline in mTSS, as well as joint erosion and narrowing scores, were also numerically lower with upadacitinib 15 mg and 30 mg relative to MTX at all radiographic assessments through week 260. Ultimately, however, both upadacitinib and MTX appeared to successfully slow joint damage progression, and there was effectively no clinically meaningful difference between the treatment arms in this regard.

The safety profile of upadacitinib over 5 years was generally consistent with earlier results from this trial and integrated phase 3 safety analyses [12, 24, 27, 29]. Most TEAEs were more frequent in patients receiving upadacitinib 30 mg compared to upadacitinib 15 mg or MTX. Moreover, patients who switched from upadacitinib 30 mg to 15 mg had similar event rates post-switch compared to those initially randomized to upadacitinib 15 mg. The rates of CPK elevation, serious infections, herpes zoster, NMSC, and neutropenia were also numerically higher with upadacitinib, especially in the 30 mg treatment group, compared to MTX. It is important to note, however, that the majority of CPK events were asymptomatic and transient; moreover, most herpes zoster events were nonserious and limited to only 1 or 2 dermatomes. Rates of MACE and VTE were comparable across treatment groups. Malignancies excluding NMSC also occurred at similar rates between upadacitinib 15 mg or 30 mg and MTX and were generally consistent with the rates expected in RA populations based on real-world data [30–32]. Rates of death were similar between upadacitinib 15 mg and MTX, but numerically higher with upadacitinib 30 mg.

Limitations of this study include potential biases inherent to LTE studies, an example of which is that they tend to overestimate treatment efficacy because patients who continue participating in the trial over the long term are typically those who respond well to the treatment and tolerate it without issues. To address this potential bias, we included more conservative estimates based on NRI in addition to AO data. Another limitation is that although most patients had a relatively short duration of RA, a proportion had been living with the disease for a longer period but had not undergone treatment with MTX. Lastly, the trial was restricted to patients who had risk factors for radiographic progression, which may not be representative of all MTX-naïve patients with RA. Thus, caution should be exercised when extrapolating these findings to other patient populations. However, despite these limitations, results from this 5-year study provide important insights into the long-term benefit-risk of upadacitinib versus MTX in a clinically controlled setting. Additionally, with a total of 945 patients treated, SELECT-EARLY stands as one of the largest, global double-blind trials ever conducted in MTX-naïve patients.

## Conclusions

In summary, upadacitinib 15 mg or 30 mg showed better clinical, radiographic, and patient-reported outcomes versus MTX in patients with RA throughout the 5-year trial. Higher rates of serious infection, herpes zoster, and neutropenia were observed with upadacitinib compared to MTX, especially in the 30 mg group; higher rates of CPK elevation was also observed with both upadacitinib doses but without clinical consequences. When used as monotherapy in MTX-naïve patients, treatment with upadacitinib 15 mg demonstrated better long-term efficacy versus MTX and an overall favorable benefit-risk profile. These findings support the use of the 15 mg daily dose for RA, as has been approved by the US Food and Drug Administration and the European Medicine Agency.

### Electronic supplementary material

Below is the link to the electronic supplementary material.

Supplementary Material 1

## Data Availability

AbbVie is committed to responsible data sharing regarding the clinical trials we sponsor. This includes access to anonymized, individual, and trial-level data (analysis data sets), as well as other information (eg, protocols, clinical study reports, or analysis plans), as long as the trials are not part of an ongoing or planned regulatory submission. This includes requests for clinical trial data for unlicensed products and indications. These clinical trial data can be requested by any qualified researchers who engage in rigorous, independent, scientific research, and will be provided following review and approval of a research proposal, Statistical Analysis Plan (SAP), and execution of a Data Sharing Agreement (DSA). Data requests can be submitted at any time after approval in the US and Europe and after acceptance of this manuscript for publication. The data will be accessible for 12 months, with possible extensions considered. For more information on the process or to submit a request, visit the following link: https://vivli.org/ourmember/abbvie/ then select “Home.”

## Abbreviations

ACPA: Anti–citrullinated protein antibodies
ACR: American College of Rheumatology
AE: Adverse event
AESI: Adverse event of special interest
AO: As observed
bDMARD: Biologic disease-modifying antirheumatic drug
CAC: Cardiovascular Advisory Committee
CDAI: Clinical Disease Activity Index
CPK: Creatine phosphokinase
csDMARD: Conventional synthetic disease-modifying antirheumatic drug
CTC: Common Toxicity Criteria
CV: Cardiovascular
DAS28(CRP): 28-joint Disease Activity Score based on C-reactive protein
DVT: Deep vein thrombosis
E: Event
EAER: Exposure-adjusted event rate
EULAR: European Alliance of Associations for Rheumatology
HAQ-DI: Health Assessment Questionnaire-Disability Index
JAK: Janus kinase
LDA: Low disease activity
LTE: Long-term extension
MACE: Major adverse cardiovascular events
MedDRA: Medical Dictionary for Regulatory Activities
MI: Myocardial
MMRM: Mixed model for repeated measures
mTSS: Modified Total Sharp Score
MTX: Methotrexate
NMSC: Nonmelanoma skin cancer
NRI: Non-responder imputation
OMERACT: Outcome Measures in Rheumatology
PY: Patient-years
RA: Rheumatoid arthritis
RF: Rheumatoid factor
TEAE: Treatment-emergent adverse event
VTE: Venous thromboembolism
