# Supplementary material for: Upadacitinib monotherapy versus methotrexate monotherapy in patients with rheumatoid arthritis: efficacy and safety through 5 years in the SELECT-EARLY randomized controlled trial
Source: Arthritis Res Ther. 2024 Jul 29;26:143. doi: 10.1186/s13075-024-03358-x (PMC11285135; doi:10.1186/s13075-024-03358-x)
Supplement: Supplementary file 1 — Supplementary Material 1 [file 13075_2024_3358_MOESM1_ESM.docx]

**SUPPLEMENTAL MATERIALS**

**Upadacitinib Monotherapy Versus Methotrexate Monotherapy in Patients With Rheumatoid Arthritis: Efficacy and Safety Through 5 Years in the SELECT-EARLY Randomized Controlled Trial**

Ronald van Vollenhoven,^1^ Vibeke Strand,^2^ Tsutomu Takeuchi,^3^ Nilmo Chávez,^4^ Pablo Mannucci Walter, ^5^ Atul Singhal,^6^ Jerzy Swierkot,^7^ Nasser Khan,^8^ Xianwei Bu,^8^ Yihan Li,^8^ Sara K. Penn,^8^ Heidi S. Camp,^8^
Jacob Aelion^9^

^1^Amsterdam University Medical Centers, Amsterdam, Netherlands

^2^Division Immunology/Rheumatology, Stanford University, Palo Alto, CA, USA

^3^Keio University School of Medicine, Tokyo, and Saitama Medical University, Saitama, Japan

^4^Instituto Guatemalteco de Seguridad Social, Ciudad de Guatemala, Guatemala

^5^Aprillus Asistencia e Investigación, Buenos Aires, Argentina

^6^Southwest Rheumatology Research Group, Dallas, TX, USA

^7^Department of Rheumatology and Internal Medicine, Wroclaw Medical University, Wroclaw, Poland

^8^AbbVie Inc., North Chicago, IL, USA

^9^West Tennessee Research Institute, Jackson, TN, USA

Address correspondence to:

Professor Ronald van Vollenhoven, MD, PhD

Amsterdam University Medical Centers, Amsterdam, Netherlands

Email: [r.vanvollenhoven@amsterdamumc.nl](mailto:r.vanvollenhoven@amsterdamumc.nl)

Phone: 31-205667765

**Major Adverse Cardiovascular Events**

A total of 10 reports of major adverse cardiovascular events (MACE) occurred in the initial monotherapy groups and include: 1 non-fatal myocardial infarction (MI), 1 sudden cardiac death, and 1 other cardiovascular (CV) death (preferred term: acute anoxic encephalopathy and MI) with upadacitinib 15 mg monotherapy; 2 sudden cardiac deaths, 1 other CV death (preferred term: cardiac failure), and 1 non-fatal MI with upadacitinib 30 mg monotherapy; 1 sudden cardiac death, 1 non-fatal MI, and 1 non-fatal stroke with methotrexate (MTX) monotherapy. One non-fatal MI also occurred in a patient who switched from upadacitinib 30 mg monotherapy to upadacitinib 15 mg monotherapy.

**Venous Thromboembolism**

A total of 12 venous thromboembolism (VTE) events occurred among the initial monotherapy groups and include 2 non-fatal pulmonary embolism (PE) and 1 non-fatal deep vein thrombosis (DVT) with upadacitinib 15 mg monotherapy; 2 non-fatal PE, 1 non-fatal DVT, and 1 concurrent case of non-fatal DVT and PE with upadacitinib 30 mg monotherapy; 2 non-fatal DVT, 2 non-fatal PE, and 1 concurrent case of non-fatal DVT and PE with MTX monotherapy. One non-fatal DVT also occurred in the upadacitinib 30 mg monotherapy switched to upadacitinib 15 mg monotherapy group.

**Hepatic Disorders**

The exposure-adjusted event rate (EAER) of treatment-emergent hepatic disorders was higher in the upadacitinib 30 mg monotherapy group (13.9 events [E]/100 patient-years [PY]) compared with the upadacitinib 15 mg monotherapy group (10.4 E/100 PY) and the methotrexate (MTX) group (10.9 E/100 PY). No Hy's law case was identified; however, 5 events (3 with upadacitinib 15 mg monotherapy and 2 with MTX monotherapy) met the numerical biochemical criteria for Hy's law. Four of these events had alternative etiologies and were not considered Hy's law cases. The remaining event occurred during a patient’s hospitalization for multi-organ failure related to acute ethanol poisoning and was also not considered a Hy’s Law case. The majority of hepatic disorder events were mild or moderate increases in alanine transferase (ALT) or aspartate transferase (AST).

**Malignancy Excluding Nonmelanoma Skin Cancer**

A total of 22 malignancies excluding nonmelanoma skin cancer (NMSC) were reported in the initial monotherapy groups. Six malignancies excluding NMSC occurred in the upadacitinib 15 mg monotherapy group, including 1 case each of breast cancer, malignant melanoma, neuroendocrine tumor, papillary thyroid cancer, squamous cell carcinoma of the lung, and uterine carcinoma in situ. Eight malignancies excluding NMSC were reported in the upadacitinib 30 mg monotherapy group, including 2 cases of cervix carcinoma (stage 0) and 1 case each of bladder cancer, gastric cancer, lung squamous cell carcinoma, ovarian germ cell teratoma, renal cancer, and uterine cancer. Eight malignancies excluding NMSC occurred in the MTX monotherapy group, including 1 case each of adenocarcinoma of the colon, anal cancer (stage 0), endometrial adenocarcinoma, lung adenocarcinoma, metastatic neoplasm, ovarian cancer, renal cell carcinoma (stage 1), and tongue neoplasm malignant. In upadacitinib + MTX/conventional synthetic disease-modifying antirheumatic drugs (csDMARDs) rescue groups, 1 malignant palate neoplasm occurred in the upadacitinib 15 mg + MTX/csDMARD group; 1 B-cell lymphoma and 1 breast cancer metastatic occurred in the upadacitinib 30 mg + MTX/csDMARDs group; 1 lung adenocarcinoma and 1 non-small cell lung cancer occurred in the upadacitinib 30 mg + MTX/csDMARDs group to upadacitinib 15 mg + MTX/csDMARD group.

**Deaths**

The rates of all deaths occurring during the study, including both treatment-emergent (ie, occurred ≤30 days after the last dose of study drug) and non-treatment-emergent (ie, occurred >30 days after the last dose of the study drug), were 0.6, 1.2, and 0.9 E/100 PY for patients in the upadacitinib 15 mg, upadacitinib 30 mg, or MTX monotherapy groups, respectively. Thirty-one total deaths were reported during the study, including 18 treatment-emergent and 13 non-treatment-emergent deaths. Of these 31 deaths, 23 deaths occurred in patients who remained on the original monotherapy treatment (with no rescue treatment or dose switching) during the study. Based on the Cardiovascular Adjudication Committee (CAC), there were 8 cardiovascular deaths (2 events in the MTX group, 3 events in the upadacitinib 15 mg group, and 3 events in the upadacitinib 30 mg group), 19 non-cardiovascular deaths, and 4 unknown/undetermined deaths. Treatment-emergent adverse events (TEAEs) leading to death include malignant melanoma, hypoxic-ischemic encephalopathy, cardiac arrest, MI, and death (unknown cause) in the upadacitinib 15 mg monotherapy group; acute respiratory failure, COVID-19 pneumonia (2), septic shock, cardiac failure, cardiovascular disorder, sudden death, peritonitis, and bladder cancer in the upadacitinib 30 mg monotherapy group. Additionally, gastric adenocarcinoma, COVID-19 pneumonia (2), and cardio-respiratory deaths were reported in the upadacitinib 30 mg monotherapy switched to 15 mg monotherapy group. Acute MI was reported in the MTX monotherapy group. In patients who received rescue therapy (ie, upadacitinib 15 mg or 30 mg + MTX/csDMARDs), other AEs leading to death were as follows: meningitis bacterial infection in the upadacitinib 30 mg + MTX/ csDMARD group. In patients who switched from upadacitinib 30 mg + MTX/csDMARDs to upadacitinib 15 mg + MTX/csDMARDs, 2 events of COVID-19 pneumonia were reported as leading to death.

**Supplemental Figure 1. Study Design of SELECT-EARLY**


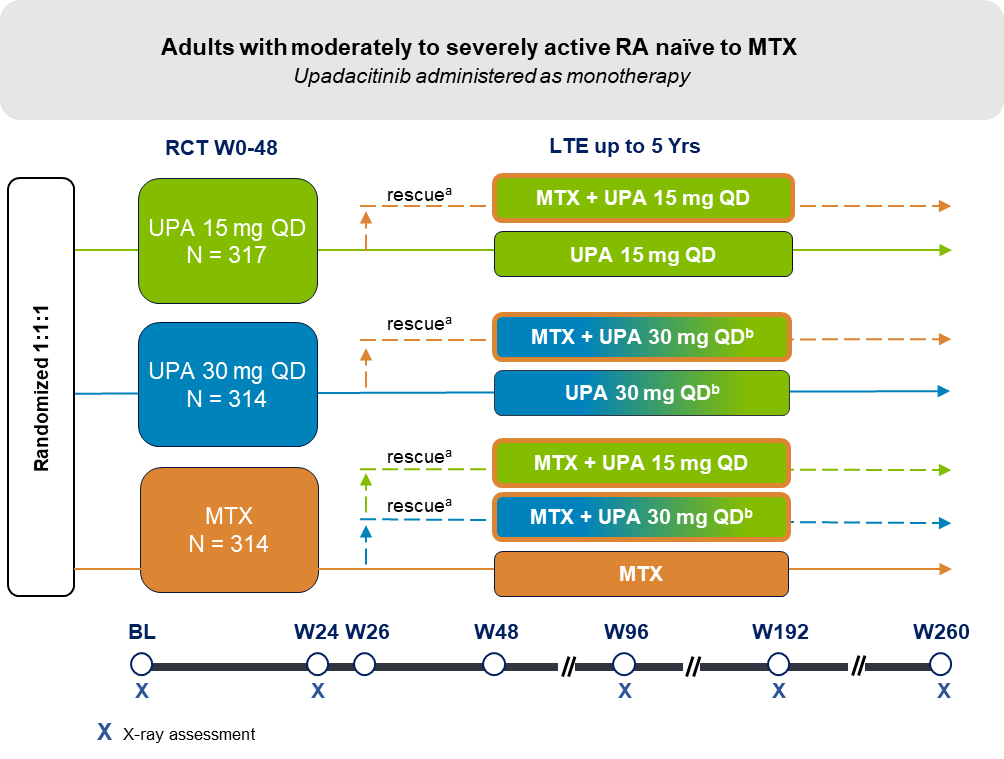


CDAI, Clinical Disease Activity Index; LTE, long-term extension; MTX, methotrexate; QD, once daily; RA; rheumatoid arthritis; RCT, randomized controlled trial; UPA, upadacitinib; W, week; yrs, years.

^a^Patients who did not achieve CDAI remission and had <20% improvement in tender and swollen joint counts at week 26 received rescue therapy (addition of MTX in the UPA groups and addition of UPA 15 mg or 30 mg in the MTX group).

^b^Patients in the UPA 30 mg treatment group were later switched to the approved UPA 15 mg dose per protocol amendment. The switch occurred at different visits across the patient population, with the earliest switch occurring at the week 108 visit.

**Supplemental Figure 2. Proportions of Patients Achieving CDAI Remission or LDA Before and After Dose Switch From Upadacitinib 30 mg to 15 mg (AO)**


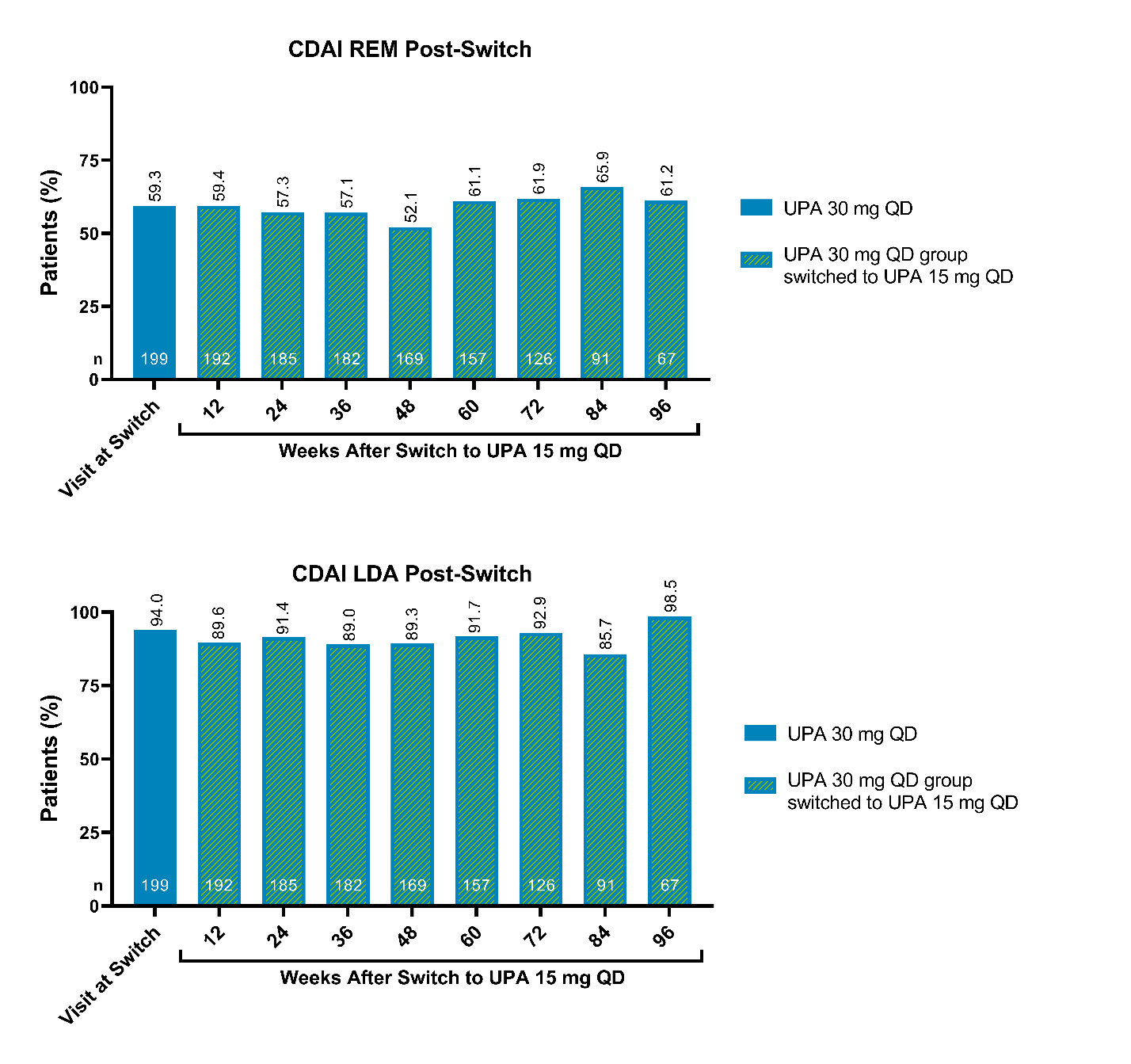


CDAI, Clinical Disease Activity Index; LDA, low disease activity; PBO, placebo; QD, once daily; REM, remission; UPA, upadacitinib.

Patients receiving UPA 30 mg treatment were switched to receiving the approved UPA 15 mg dose per later protocol amendment. The switch occurred at different visits across the patient population, with the earliest switch occurring at the week 108 visit.

**Supplemental Figure 3. Mean Change From Baseline Through 5 Years in Core Components of the ACR Response Criteria (AO, MMRM)**

**
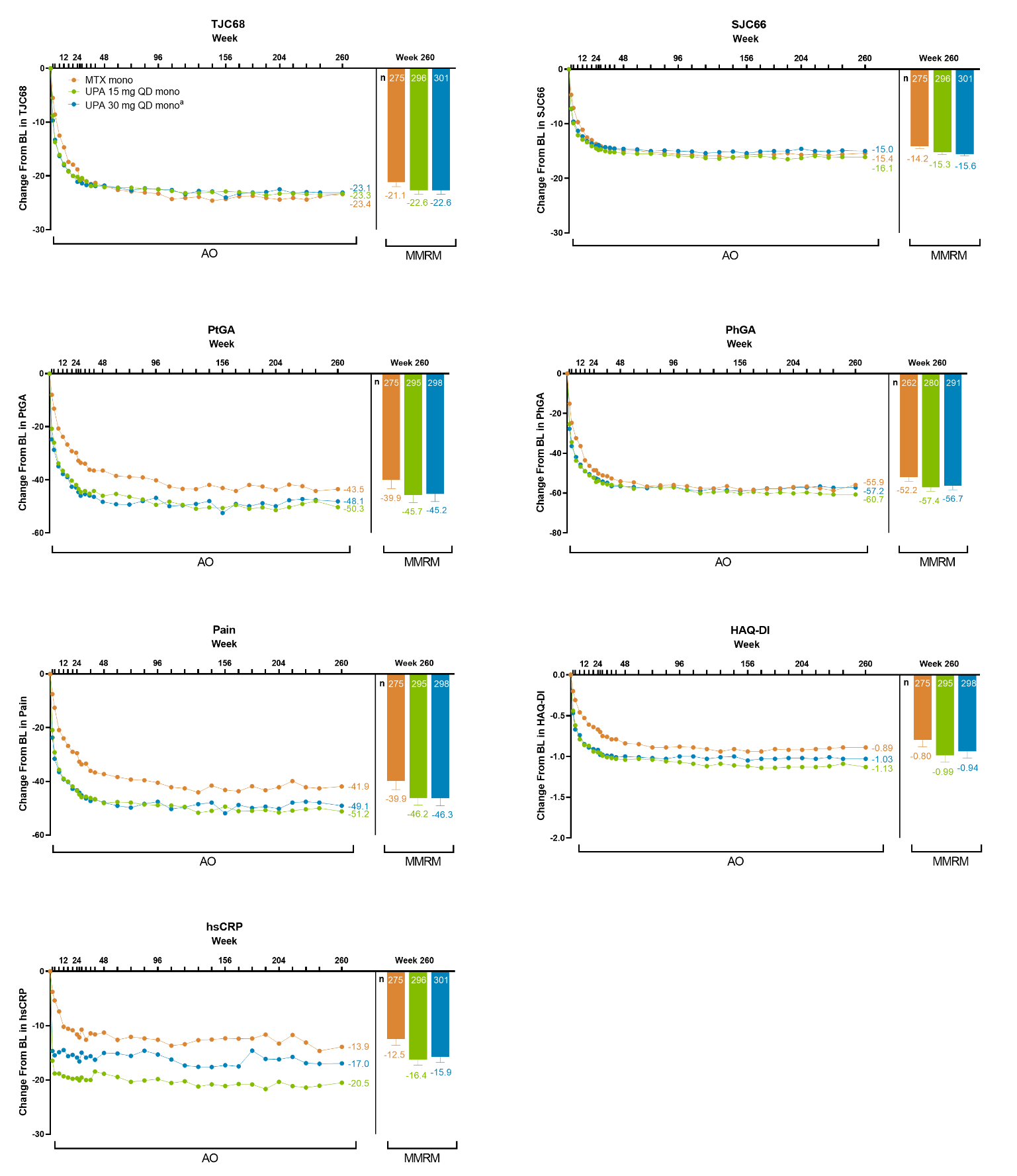
**

ACR, American College of Rheumatology; AO, as observed; BL, baseline; HAQ-DI, Health Assessment Questionnaire Disability-Index; hsCRP, high-sensitivity C-reactive protein; mono, monotherapy; MMRM, mixed model for repeated measures; MTX, methotrexate; PhGA, physician’s global assessment of disease activity; PtGA, patient’s global assessment of disease activity; QD, once daily; SJC, swollen joint count; TJC, tender joint count; UPA, upadacitinib.

^a^Patients in the UPA 30 mg treatment group were later switched to the approved UPA 15 mg dose per protocol amendment. The switch occurred at different visits across the patient population, with the earliest switch occurring at the week 108 visit.

Error bars represent the 95% confidence interval.

**Supplemental Figure 4. Mean Change From Baseline Through 5 Years in Morning Stiffness Severity and Duration (AO, MMRM)**


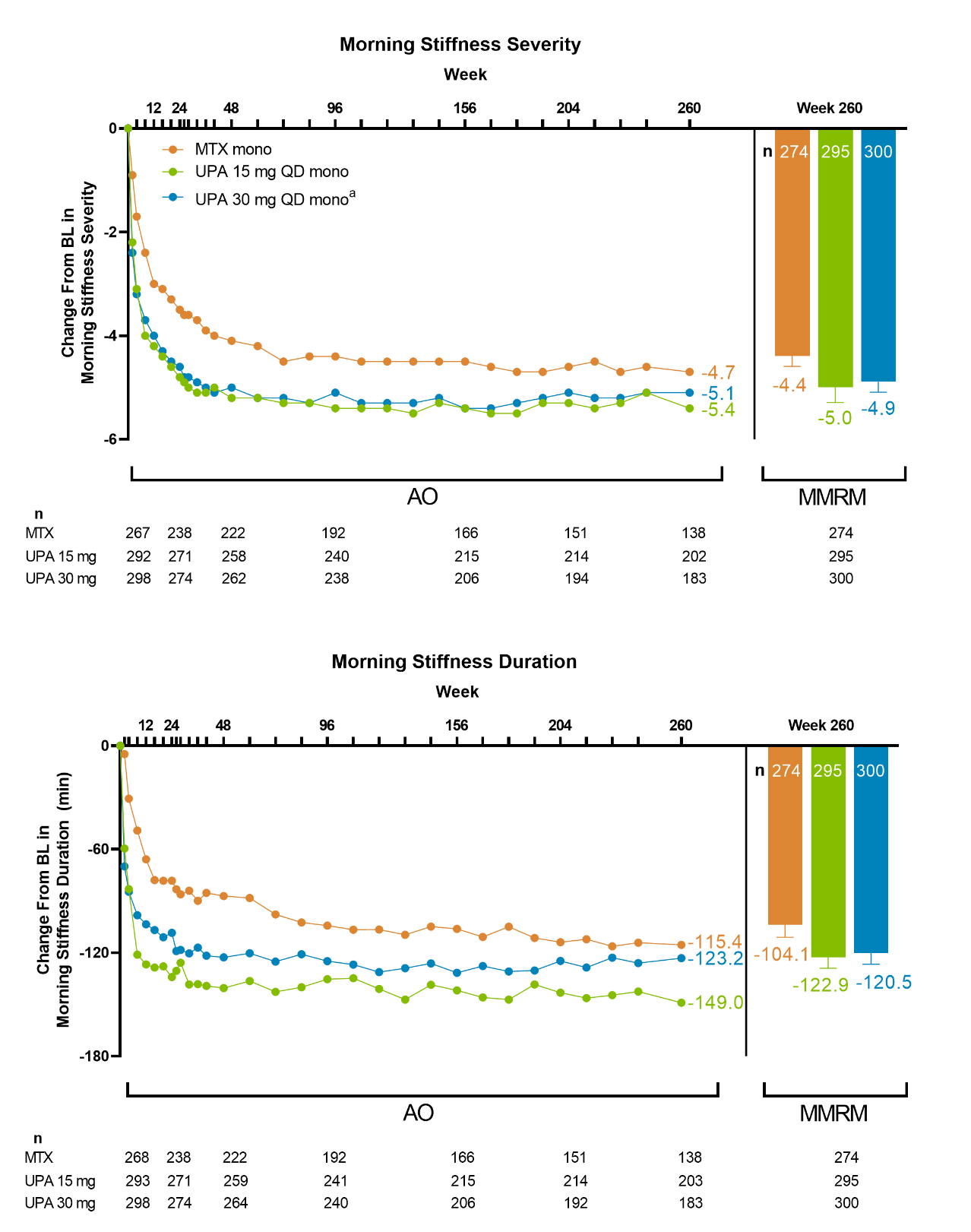


AO, as observed; BL, baseline; MMRM, mixed model for repeated measures; mono, monotherapy; MTX, methotrexate; QD, once daily; UPA, upadacitinib.

^a^Patients in the UPA 30 mg treatment group were later switched to the approved UPA 15 mg dose per protocol amendment. The switch occurred at different visits across the patient population, with the earliest switch occurring at the week 108 visit.

Morning stiffness was assessed as change from BL on a 0–10 numerical rating scale ranging from “no morning stiffness at all” to “extremely severe morning stiffness.” Error bars represent the 95% confidence interval.

| **Supplemental Table 1. Baseline Demographics and Disease Characteristics of Patients in SELECT-EARLY**   \| **Characteristic^a^** \| **MTX**  **(N = 314)** \| **UPA 15 mg QD**  **(N = 317)** \| **UPA 30 mg QD**  **(N = 314)** \| \| --- \| --- \| --- \| --- \| \| Time since RA diagnosis, years \| 2.6 ± 5.1 \| 2.9 ± 5.4 \| 2.8 ± 5.6 \| \| Median (range), years \| 0.5 (0.0–38.0) \| 0.5 (0.0–36.5) \| 0.6 (0.4–44.0) \| \| Female, no. (%) \| 240 (76.4) \| 241 (76.0) \| 240 (76.4) \| \| Age, years \| 53.3 ± 12.9 \| 51.9 ± 12.6 \| 54.9 ± 12.6 \| \| Previous csDMARD exposure, no. (%) \| 79 (25.2) \| 80 (25.2) \| 80 (25.5) \| \| MTX exposure, no. (%) \| 19 (6.1) \| 30 (9.5) \| 22 (7.0) \| \| MTX dose at week 24, mg \| 19.2 ± 2.1 \| – \| – \| \| Race, no. (%) \|  \|  \|  \| \| American Indian or Alaska Native \| 2 (0.6) \| 8 (2.5) \| 7 (2.2) \| \| Asian \| 37 (11.8) \| 35 (11.0) \| 34 (10.8) \| \| Black or African American \| 12 (3.8) \| 8 (2.5) \| 13 (4.1) \| \| Multiple \| 5 (1.6) \| 7 (2.2) \| 5 (1.6) \| \| Native Hawaiian or other Pacific Islander \| 2 (0.6) \| 3 (0.9) \| 1 (0.3) \| \| White \| 256 (81.5) \| 256 (80.8) \| 254 (80.9) \| \| Oral glucocorticoid use, no. (%) \| 163 (51.9) \| 147 (46.4) \| 137 (43.6) \| \| Oral glucocorticoid dose, mg/day^b^ \| 6.4 ± 2.4 \| 6.4 ± 3.1 \| 6.9 ± 2.9 \| \| RF and/or ACPA positive, no. (%) \| 255 (81.2) \| 279 (88.3) \| 252 (80.5) \| \| TJC68 \| 26.4 ± 16.2 \| 25.4 ± 14.4 \| 25.2 ± 15.0 \| \| SJC66 \| 16.9 ± 10.6 \| 16.9 ± 10.4 \| 15.7 ± 9.7 \| \| PtGA (0–100 mm VAS)^c^ \| 65.8 ± 21.5 \| 66.6 ± 22.0 \| 64.9 ± 21.6 \| \| PhGA (0–100 mm VAS)^d^ \| 68.7 ± 16.5 \| 67.1 ± 17.0 \| 65.3 ± 16.6 \| \| Pain (0–100 mm VAS)^c^ \| 65.7 ± 21.5 \| 68.4 ± 20.6 \| 65.3 ± 21.5 \| \| hsCRP, mg/L \| 21.2 ± 22.1 \| 23.0 ± 27.4 \| 19.4 ± 22.6 \| \| DAS28(CRP)^c^ \| 5.9 ± 1.0 \| 5.9 ± 1.0 \| 5.8 ± 1.0 \| \| CDAI^e^ \| 40.5 ± 13.3 \| 40.4 ± 13.3 \| 39.3 ± 13.5 \| \| SDAI^e^ \| 42.6 ± 14.0 \| 42.7 ± 13.9 \| 41.3 ± 14.4 \| \| mTSS^f^ \| 13.3 ± 30.6 \| 18.1 ± 38.2 \| 17.2 ± 38.3 \| \| Erosion score^f^ \| 6.1 ± 15.5 \| 8.6 ± 19.3 \| 8.0 ± 18.9 \| \| JSN score^f^ \| 7.2 ± 16.2 \| 9.6 ± 20.1 \| 9.3 ± 20.3 \| \| Morning stiffness \|  \|  \|  \| \| Duration, minutes^g^ \| 128.5 ± 134.2 \| 168.9 ± 227.5 \| 136.4 ± 166.5 \| \| Severity (0–10 scale)^g^ \| 6.3 ± 2.3 \| 6.6 ± 2.3 \| 6.4 ± 2.2 \| \| HAQ-DI^c^ \| 1.6 ± 0.7 \| 1.6 ± 0.7 \| 1.5 ± 0.7 \| \| FACIT‐F^h^ \| 26.6 ± 11.7 \| 26.4 ± 11.9 \| 27.8 ± 11.1 \| \| SF‐36 PCS^i^ \| 33.1 ± 7.5 \| 32.7 ± 7.7 \| 33.7 ± 7.2 \| |
| --- | --- | --- | --- | --- | --- | --- | --- | --- | --- | --- | --- | --- | --- | --- | --- | --- | --- | --- | --- | --- | --- | --- | --- | --- | --- | --- | --- | --- | --- | --- | --- | --- | --- | --- | --- | --- | --- | --- | --- | --- | --- | --- | --- | --- | --- | --- | --- | --- | --- | --- | --- | --- | --- | --- | --- | --- | --- | --- | --- | --- | --- | --- | --- | --- | --- | --- | --- | --- | --- | --- | --- | --- | --- | --- | --- | --- | --- | --- | --- | --- | --- | --- | --- | --- | --- | --- | --- | --- | --- | --- | --- | --- | --- | --- | --- | --- | --- | --- | --- | --- | --- | --- | --- | --- | --- | --- | --- | --- | --- | --- | --- | --- | --- | --- | --- | --- | --- | --- | --- | --- | --- | --- | --- | --- | --- | --- | --- | --- | --- | --- | --- | --- | --- | --- | --- | --- | --- | --- | --- | --- | --- | --- | --- | --- |

Reprinted from *Arthritis Rheumatol*, Vol. 72, van Vollenhoven R et al, Efficacy and Safety of Upadacitinib Monotherapy in Methotrexate-Naive Patients With Moderately-to-Severely Active Rheumatoid Arthritis (SELECT-EARLY): A Multicenter, Multi-Country, Randomized, Double-Blind, Active Comparator-Controlled Trial, Pages 1607-1620. Copyright (2020), with permission from John Wiley and Sons.

ACPA, anti–citrullinated protein antibodies; csDMARD, conventional synthetic disease‐modifying antirheumatic drug; DAS28(CRP), Disease Activity Score in 28 joints using the CRP level; FACIT‐F, Functional Assessment of Chronic Illness Therapy–Fatigue scale; HAQ-DI, Health Assessment Questionnaire-Disability Index; hsCRP, high‐sensitivity C‐reactive protein; JSN, joint space narrowing; mTSS, modified Total Sharp Score; PhGA, physician global assessment of disease activity; PtGA, patient global assessment of disease activity; RA, rheumatoid arthritis; RF, rheumatoid factor; SF‐36 PCS, Short Form 36 physical component summary; VAS, visual analog scale.

^a^Except where indicated otherwise, values are the mean ± SD. Percentages were calculated based on non-missing values.

^b^Based on prednisone equivalent dose. Only patients who were receiving oral steroids at baseline were evaluated.

^c^MTX, n=314; UPA 15 mg, n=317; UPA 30 mg, n=311.

^d^MTX, n=299; UPA 15 mg, n=301; UPA 30 mg, n=304.

^e^MTX, n=299; UPA 15 mg, n=301; UPA 30 mg, n=303.

^f^MTX, n=309; UPA 15 mg, n=309; UPA 30 mg, n=309.

^g^MTX, n=313; UPA 15 mg, n=316; UPA 30 mg, n=313.

^h^MTX, n=314; UPA 15 mg, n=316; UPA 30 mg, n=310.

^i^MTX, n=313; UPA 15 mg, n=315; UPA 30 mg, n=312.

**Supplemental Table 2. Most Common Treatment-Emergent Adverse Events (≥5 E/100 PY in Any Treatment Group) Through 5 Years**

| **Events (E/100 PY)^a^** | **MTX mono  (N = 314; PY = 860.2)** | **UPA 15 mg QD mono (N = 317; PY = 1062.6)** | **UPA 30 mg QD mono^b^**  **(N = 314; PY = 741.5)** | **UPA 15 mg QD mono switched from UPA 30 mg QD mono**  **(N = 181; PY = 292.5)** |
| --- | --- | --- | --- | --- |
| Blood creatine phosphokinase increased | 12 (1.4) | 68 (6.4) | 106 (14.3) | 14 (4.8) |
| Upper respiratory tract infection | 45 (5.2) | 88 (8.3) | 75 (10.1) | 7 (2.4) |
| Urinary tract infection | 59 (6.9) | 93 (8.8) | 67 (9.0) | 16 (5.5) |
| Nasopharyngitis | 70 (8.1) | 87 (8.2) | 72 (9.7) | 10 (3.4) |
| Neutropenia | 15 (1.7) | 26 (2.4) | 37 (5.0) | 9 (3.1) |
| Alanine aminotransferase increased | 34 (4.0) | 49 (4.6) | 48 (6.5) | 4 (1.4) |
| Hypertension | 35 (4.1) | 40 (3.8) | 46 (6.2) | 8 (2.7) |
| Bronchitis | 28 (3.3) | 27 (2.5) | 39 (5.3) | 9 (3.1) |
| Nausea | 47 (5.5) | 28 (2.6) | 19 (2.6) | 1 (0.3) |
| Rheumatoid arthritis | 65 (7.6) | 37 (3.5) | 12 (1.6) | 19 (6.5) |
| COVID-19 | 20 (2.3) | 28 (2.6) | 4 (0.5) | 22 (7.5) |

Mono, monotherapy; MTX, methotrexate; PY, patient-years; QD, once daily; TEAE, treatment-emergent adverse event; UPA, upadacitinib.

^a^Data are presented as treatment-emergent adverse events, which is defined as any adverse event with an onset date that is after the first dose of study drug and no more than 30 days after the last dose of study drug. Data include patients receiving UPA or MTX monotherapy, censored at either time of rescue to UPA + MTX or with addition of background conventional synthetic DMARD.

^b^UPA 30 mg exposure was censored at the time of dose switch to the approved 15 mg dose. Safety outcomes following the switch from UPA 30 mg to UPA 15 mg are reported separately (last column).

**Supplemental Table 3. Grade 3/4 Laboratory Abnormalities Through 5 Years**

|  |
| --- |

**Supplemental Table 3. Grade 3/4 Laboratory Abnormalities Through 5 Years**

| \| **Parameter^a^** \| \| **MTX mono**  **(N = 314)**  **n/N_obs ^b^ (%)** \| **UPA 15 mg QD mono**  **(N = 317)**  **n/N_obs ^b^  (%)** \| **UPA 30 mg QD mono^c^**  **(N = 314)**  **n/N_obs ^b^ (%)** \| **UPA 15 mg QD mono switched from UPA 30 mg QD mono**  **(N = 181)**  **n/N_obs ^b^ (%)** \| \| --- \| --- \| --- \| --- \| --- \| --- \| \| Hemoglobin (g/dL) \| Grade 3 (decreased 2.1 to < 3.0^d^ or Hb ≥ 7.0 to < 8.0) \| 32/312 (10.3) \| 28/315 (8.9) \| 46/311 (14.8) \| 14/173 (8.1) \| \| Grade 4 (decreased  ≥ 3.0^d^  or Hb < 7.0) \| 23/312 (7.4) \| 14/315 (4.4) \| 19/311 (6.1) \| 8/173 (4.6) \| \| Lymphocytes  (× 10^9^/L) \| Grade 3 (0.5 to < 1.0) \| 87/312 (27.9) \| 94/315 (29.8) \| 105/310 (33.9) \| 70/173 (40.5) \| \| Grade 4 (< 0.5) \| 7/312 (2.2) \| 7/315 (2.2) \| 9/310 (2.9) \| 3/173 (1.7) \| \| Neutrophils  (× 10^9^/L) \| Grade 3 (0.5 to < 1.0) \| 3/312 (1.0) \| 8/315 (2.5) \| 11/310 (3.5) \| 3/173 (1.7) \| \| Grade 4 (< 0.5) \| 1/312 (0.3) \| 1/315 (0.3) \| 1/310 (0.3) \| 0/173 (0) \| \| ALT (U/L) \| Grade 3 (3.0 to < 8.0 × ULN) \| 26/312 (8.3) \| 16/315 (5.1) \| 18/310 (5.8) \| 6/176 (3.4) \| \| Grade 4 (> 8.0 × ULN) \| 6/312 (1.9) \| 6/315 (1.9) \| 2/310 (0.6) \| 0/176 (0) \| \| AST (U/L) \| Grade 3 (3.0 to 8.0 × ULN) \| 16/312 (5.1) \| 14/315 (4.4) \| 12/310 (3.9) \| 1/176 (0.6) \| \| Grade 4 (> 8.0 × ULN) \| 2/312 (0.6) \| 4/315 (1.3) \| 2/310 (0.6) \| 0/176 (0) \| \| CPK (U/L) \| Grade 3 (> 5.0 × ULN to 10.0 × ULN) \| 3/312 (1.0) \| 7/315 (2.2) \| 9/311 (2.9) \| 1/174 (0.6) \| \| Grade 4 (> 10.0 × ULN) \| 0/312 \| 4/315 (1.3) \| 7/311 (2.3) \| 1/174 (0.6) \| \| Creatinine (μMol/L) \| Grade 3 (> 3.0 to 6.0 × ULN) \| 0/312 (0) \| 0/315 (0) \| 0/312 (0) \| 0/176 (0) \| \| Grade 4 (> 6.0 × ULN) \| 0/312 (0) \| 2/315 (0.6) \| 0/312 (0) \| 0/176) (0) \| |
| --- | --- | --- | --- | --- | --- | --- | --- | --- | --- | --- | --- | --- | --- | --- | --- | --- | --- | --- | --- | --- | --- | --- | --- | --- | --- | --- | --- | --- | --- | --- | --- | --- | --- | --- | --- | --- | --- | --- | --- | --- | --- | --- | --- | --- | --- | --- | --- | --- | --- | --- | --- | --- | --- | --- | --- | --- | --- | --- | --- | --- | --- | --- | --- | --- | --- | --- | --- | --- | --- | --- | --- | --- | --- | --- | --- | --- | --- | --- | --- | --- | --- | --- | --- |

ALT, alanine aminotransferase; AST, aspartate aminotransferase; CPK, creatine phosphokinase; Hb, hemoglobin; mono, monotherapy; MTX, methotrexate; QD, once daily; ULN, upper limit of the normal range; UPA, upadacitinib.

^a^Data are for patients with worsening in grade severity for laboratory parameters. Grading was based on Outcome Measures in Rheumatology (OMERACT) criteria, except for CPK and creatinine, where the National Cancer Institute’s standard common terminology criteria was used. Data include patients receiving UPA or MTX monotherapy, censored at either time of rescue to UPA + MTX or with addition of background conventional synthetic DMARD.

^b^N_obs indicates the number of patients with baseline and post-baseline values for the respective parameter.

^c^UPA 30 mg exposure was censored at the time of dose switch to the approved 15 mg dose. Safety outcomes following the switch from UPA 30 mg to UPA 15 mg are reported separately (last column).

^d^Decrease from baseline. Baseline is defined as the last observation on or before the date of the first dose of study drug in the corresponding treatment group.

|  |
| --- |
